# Editorial: Challenging standards and paradigms to support animal disease prevention and control

**DOI:** 10.3389/fvets.2023.1208023

**Published:** 2023-05-23

**Authors:** Brian D. Perry, Karl M. Rich, Andres M. Perez

**Affiliations:** ^1^Nuffield College of Clinical Medicine, University of Oxford, Oxford, United Kingdom; ^2^College of Medicine and Veterinary Medicine, University of Edinburgh, Edinburgh, United Kingdom; ^3^Department of Agricultural Economics, Ferguson College of Agriculture, Oklahoma State University, Stillwater, OK, United States; ^4^Department of Veterinary Population Medicine, College of Veterinary Medicine, University of Minnesota, St Paul, MN, United States

**Keywords:** international organizations, standards, trade, epidemiology, animal disease

Despite progress made in the control of many animal diseases, and on the development of science-based solutions to mitigate the potential impacts of current and emerging health challenges, much work still needs to be done to exploit the impacts of this progress on global animal health. A particular gap is in understanding the contextual drivers that underpin and influence the dynamics of animal disease outbreaks and complicate their control. Accordingly, there is a strong need for synergistic, transdisciplinary teams to address these socio-political and economic aspects of disease risk, dynamics, and impacts.

Alternative ideas and approaches are required to better understand the ways in which the animal health system may be modified and effected to reduce the risk for disease and promote health. Intergovernmental organizations with a mission relevant to animal health and production, such as the World Organization for Animal Health (WOAH), the WHO, and the Food and Agriculture Organization (FAO) recognize the need for improving the use and application by relevant stakeholders of epidemiology and social sciences tools (including, for example, diagnostic, data analysis and risk assessments, and communication) with the ultimate goal of managing zoonotic and high impact diseases of animals and humans. Additionally, the need for following and implementing science-based approaches is recognized by the three organizations in a variety of documents, such as the tripartite concept note on sharing responsibilities and coordinating global activities to address health risks at the animal-human-ecosystems interfaces (1).

Science is dynamic in nature, and, for that reason, there is a need to regularly review and revise standards, and to account for advancements and new scientific developments. Intergovernmental organizations recognize such need and have developed strategies to regularly update guidelines, recommendations, and standards. For example, WOAH (funded as OIE)'s scientific commission for animal diseases was created at the organization's inception in 1946, “to provide scientific guidance to the OIE on the development of policies relating to the assessment and control of diseases, notably those with the potential to affect trade in terrestrial animals and their products or affect human health.”

The collection of papers presented here displays a number of examples aimed at exploring, assessing, and proposing actions and policy that challenge current standards, and propose alternative views, on animal disease prevention and control.

Three papers explored and proposed alternative measures and policies to support the prevention and control of African Swine Fever (ASF), a disease that has continued to spread globally, reaching pandemic proportions, since 2007, causing a devastating impact to the economy of affected countries. Groenendaal et al. proposed the application of a risk analysis approach to identify the weakest links in biosecurity and design the corresponding mitigation efforts. The approach was intended to create a mechanism to work with producers and practitioners in enhancing their understanding of the potential pathways of introduction and spread specific to individual farms, with the ultimate goal of encouraging producers to invest in biosecurity measures as a strategy to mitigate the risk for disease spread beyond imposed regulations. Nga et al. explored the consequences of partial culling in Vietnam, providing, for the first time, metrics of the probability of survival of susceptible animals in an ASF-infected population. Costard et al. proposed the application of an alternative framework for ASF elimination, to incentivize producers to invest in early detection and reporting. The authors argued that the approach would promote the alignment between industry-led efforts and an appropriate and effective regulatory framework, mitigating the impact of disease.

Three papers used mixed qualitative and quantitative methods to identify areas of intervention for the improvement of disease surveillance and prevention strategies in eastern Africa. These contributions are important because they demonstrated the practical application of applied socio-economics and epidemiology to identify areas of intervention to improve the animal health system. George, Häsler, Komba, Rweyemamu et al. used a mixed-method model to evaluate the national animal health surveillance system of Tanzania in terms of fidelity, adherence, exposure, satisfaction, participation rate, recruitment and context. The authors argue that the system may benefit from the development of a user-friendly unified reporting system, the active involvement of subnational level animal health officials, optimization of data sources and an increase in the horizon of the financing mechanism. As a follow up study, George, Häsler, Komba, Sindato et al. proposed a qualitative method that assesses the relationship between existing stakeholder collaborations and influences at the subnational level to determine potential leverage points for improving the national animal health surveillance system. Results showed that animal health practitioners had a stronger relationship with community stakeholders compared to other categories of society, and contributed to map the opportunities for intervention in the system, including potential mechanisms for incentivizing the application of good practices, with the overall objective of enhancing animal disease surveillance capabilities in the country. Finally, Moje et al. conducted a cross-sectional survey to determine the biosecurity status of dairy farms and investigate the impact of socio-demographic characteristics on dairy farm management in Ethiopia. The participatory approach used here showed that biosecurity was poor in almost 80% of the dairy farms assessed, and this was significantly associated with social variables such as gender, education level, and training of the farmers. These results identified areas of intervention to improve the adoption of biosecurity practices in dairy farms of the country.

Two papers explored the role, importance, and impact that standardization of procedures has on facilitating regional trade and on the development of new technology and approaches to support animal health. Loria et al. reviewed the evolution of animal trade regulations in Europe. The paper described the progress European countries have made in harmonizing animal health standards and trade. Because of the important roles that veterinarians play in the implementation of the law, the authors argued that building the capacity of the veterinary workforce will be critically important in supporting animal health status in the region. Arnouts et al. critically reviewed and revised the use of the Technology Readiness Level (TRL) in monitoring and evaluating the development of new technologies. They aligned innovation pipeline stages as used in the animal health industry for the development of new vaccines or drugs with the TRL scale, resulting in TRL for animal health (TRLAH). The TRLAH is thus proposed as an instrument to enhance the translation of public research results into industrial and societal innovation and foster public-private partnerships in animal health.

In summary, the Research Topic here offers a collection of papers that critically assess and challenge paradigms for disease control, providing alternative views for policy and management, with the ultimate goal of supporting improved disease prevention and control at local, regional, and global scales.

## Author contributions

All authors listed have made a substantial, direct, and intellectual contribution to the work and approved it for publication.
